# Pupil Dilation Is Sensitive to Semantic Ambiguity and Acoustic Degradation

**DOI:** 10.1177/2331216520964068

**Published:** 2020-10-30

**Authors:** Mason Kadem, Björn Herrmann, Jennifer M. Rodd, Ingrid S. Johnsrude

**Affiliations:** 1 Department of Psychology, The University of Western Ontario, London, Ontario, Canada; 2 School of Biomedical Engineering, McMaster University, Hamilton, Ontario, Canada; 3 Rotman Research Institute, Baycrest, Toronto, Ontario, Canada; 4 Department of Psychology, University of Toronto, Toronto, Ontario, Canada; 5 Department of Experimental Psychology, University College London, London, United Kingdom; 6 School of Communication and Speech Disorders, The University of Western Ontario, London, Ontario, Canada

**Keywords:** pupillometry, listening effort, cognitive load, semantic ambiguity, speech masking

## Abstract

Speech comprehension is challenged by background noise, acoustic interference, and linguistic factors, such as the presence of words with more than one meaning (homonyms and homophones). Previous work suggests that homophony in spoken language increases cognitive demand. Here, we measured pupil dilation—a physiological index of cognitive demand—while listeners heard *high-ambiguity* sentences, containing words with more than one meaning, or well-matched *low-ambiguity* sentences without ambiguous words. This semantic-ambiguity manipulation was crossed with an acoustic manipulation in two experiments. In Experiment 1, sentences were masked with 30-talker babble at 0 and +6 dB signal-to-noise ratio (SNR), and in Experiment 2, sentences were heard with or without a pink noise masker at –2 dB SNR. Speech comprehension was measured by asking listeners to judge the semantic relatedness of a visual probe word to the previous sentence. In both experiments, comprehension was lower for high- than for low-ambiguity sentences when SNRs were low. Pupils dilated more when sentences included ambiguous words, even when no noise was added (Experiment 2). Pupil also dilated more when SNRs were low. The effect of masking was larger than the effect of ambiguity for performance and pupil responses. This work demonstrates that the presence of homophones, a condition that is ubiquitous in natural language, increases cognitive demand and reduces intelligibility of speech heard with a noisy background.

Following and understanding one particular conversational partner, despite interference from other sources, is a feat most of us accomplish effortlessly every day. However, many processes are required to analyze a complex auditory signal, consisting of many different sound sources, so that one source (i.e., a voice) can be identified, tracked, and understood. The process is complicated by the enormous variability of speech—speech is often in an unfamiliar accent and/or voice, distorted or degraded, or masked by other sounds. Different acoustic challenges may require different cognitive resources for speech comprehension to be successful. For example, when speech is masked energetically (i.e., by a sound with frequency components that excite the same neurons in the auditory periphery as the target; energetic masking: Schneider et al., 2007; Shinn-Cunningham, 2008), some of the speech signal is obliterated, and missing information must be inferred from the bits of speech that are perceived. This probably requires effective working memory and access to semantic knowledge (Johnsrude & Rodd, 2016). In contrast, a competing voice may be acoustically different enough from the target speech signal that energetic masking is minimal, but this still requires cognitive control and distracter suppression to not mistake (speech-like) maskers for the target speech (Johnsrude & Rodd, 2016). This is a form of *informational* masking, so called because the interference is due to the perceptual similarity between target and masker, and not to energetic masking (Durlach et al., 2003; Kidd et al., 2008; Schneider et al., 2007).

Linguistic factors also challenge speech comprehension (Gibson, 1998; Gibson & Pearlmutter, 1998). Sometimes utterances are simple and straightforward, such as the statement ‘The dog yapped at the squirrel’, but other times, the linguistic structure is more complex (‘It was the squirrel at which the dog yapped’), or the utterance lacks clear (to the listener) meaningfulness at the word and/or sentence level that would aid comprehension, because words have multiple meanings, or are uncommon (‘The bark ruffled the sciurid’). Again, the cognitive resources recruited to compensate for such linguistic demands probably differ, depending on the demand (Gibson, 1998; Johnsrude & Rodd, 2016; Van Hedger & Johnsrude, in press).

Speech understanding can be particularly challenging for those with hearing loss. Substantially greater demands must be placed on cognitive, compensatory processes in hearing-impaired individuals, who report listening in such situations to be effortful (Hornsby, 2013; Nachtegaal et al., 2009). This listening effort is a serious obstacle to communication, affecting all aspects of a person’s life (Banh et al., 2012; Pichora-Fuller et al., 2016). Listening effort is therefore increasingly recognized as a useful concept to understand the hearing problems many normally aging adults experience in their everyday lives (Eckert et al., 2016; Johnsrude & Rodd, 2016; Lemke & Besser, 2016; Peelle, 2018; Pichora-Fuller et al., 2016; Strauss & Francis, 2017; Winn et al., 2018). Listening effort may explain variance in behavior that is not captured by standard hearing assessment (e.g., audiometry). Measuring listening effort effectively has thus become a major endeavor in the hearing science and audiology communities.

Subjective ratings are a common way to assess listening effort (Alhanbali et al., 2017; Gatehouse & Noble, 2004; Krueger et al., 2017; Larsby et al., 2005; Wendt et al., 2016). However, subjective measures have a host of limitations such as context effects (participants may rate their experienced effort relative to different conditions within an experiment rather than in absolute terms of their experience) and intersubject differences in scale use. Moreover, established scales are only appropriate for use with older children and adults; nonhuman animals and babies cannot provide subjective effort ratings, and *effort* may be conceptualized differently in different cultures, limiting comparative research. Objective, physiological measures can also provide a window onto listening effort. Pupillometry—the measurement of the dilation of an individual’s pupil—has long been used to study *mental effort* (Beatty, 1982; Kahneman & Beatty, 1966; Kramer et al., 1997; Sirois & Brisson, 2014). This approach has, more recently, sparked great interest among hearing scientists and audiologists because of its potential applicability in the clinic as a way to understand cognitive demands during speech processing (Schmidtke, 2018; Winn et al., 2018; Zekveld et al., 2018).

Pupillometry studies focusing on acoustic challenges during listening demonstrate that the pupil is typically larger when individuals listen to acoustically degraded speech compared with acoustically less degraded speech (Borghini & Hazan, 2018; Miles et al., 2017; Wendt et al., 2016; Winn et al., 2015; Zekveld et al., 2010, 2014), although pupil dilation may reach an asymptote for highly degraded and cognitively demanding, but intelligible speech signals (Ohlenforst et al., 2017; Zekveld & Kramer, 2014; Zekveld et al., 2019).

We have long known that any challenge that increases the brain’s processing load will dilate the pupil (Kahneman, 1973; Kahneman & Beatty, 1966), but pupillometry has not been used very often to study the effects of linguistic challenges on speech comprehension. Two studies have shown that pupil dilation is enhanced for syntactically complex, object-first sentences compared with less complex, subject-first sentences (Ayasse & Wingfield, 2018; Wendt et al., 2016), indicating that pupillometry can provide a window onto linguistic challenges during speech comprehension.

The effect of semantic ambiguity on pupil dilation during sentence comprehension is less clear, although other work suggests that the presence of semantically ambiguous words is cognitively demanding (Johnsrude & Rodd, 2016; Rodd, in press; Rodd et al., 2005, 2010a). Indeed, isolated words that are semantically difficult to process (based on word frequency, familiarity, and other factors; Chapman & Hallowell, 2015) or words presented under lexical competition (Kuchinsky et al., 2013) lead to larger pupil dilation compared with words that are semantically easier to process. Moreover, sentences with weak semantic constraints have been shown to lead to larger pupil dilation compared with sentences with strong semantic constraints (Winn, 2016). However, sentences whose meaning is unambiguous but which contain multiple ambiguous words (e.g., The **shell** was **fired** towards the **tank**) are common in real life. In such sentences, each ambiguous word on its own is semantically consistent with a wider set of interpretations, and the overall meaning of the sentence (which is not ambiguous) depends on the constraints imposed mutually across all the ambiguous words in the sentence (shell…fired…tank) and not on any one word perceived in isolation. It is unknown whether pupillometry is sensitive to the demands imposed by such sentences.

Acoustic and linguistic challenges may interact in their effect on pupil dilation: The effect of linguistic challenges may be particularly prominent under high compared with low acoustic challenges (Kuchinsky et al., 2013; Wendt et al., 2016). In contrast, high cognitive load may cause pupil dilation to approach an asymptote (Ohlenforst et al., 2017; Zekveld & Kramer, 2014; Zekveld et al., 2019) such that acoustic and linguistic challenges may be subadditive in their effects on pupil dilation.

In a typical pupillometry study, participants are instructed to maintain fixation and reduce blinks during recordings (Wendt et al., 2016; Zekveld et al., 2019). Microsaccades commonly occur during fixation (Engbert, 2006; Martinez-Conde et al., 2009, 2013; Widmann et al., 2014) and can influence pupil dilation (Knapen et al., 2016). Microsaccade rate has also been shown to decrease with high cognitive load (Dalmaso et al., 2017; Xue et al., 2017) and task difficulty (Siegenthaler et al., 2014) and may thus reflect a potential physiological measure of cognitive demands during speech listening, but this has not been explored.

In the current study, we conducted two experiments to investigate whether semantic ambiguity and speech clarity affect sentence comprehension, pupil dilation, and microsaccade rate. In both experiments, we presented sentences containing words with more than one meaning such as ‘the **shell** was **fired** towards the **tank**’ and control sentences that were syntactically matched but did not contain ambiguous words (Rodd et al., 2005, 2010a). In Experiment 1, sentences were presented in an ongoing multitalker background babble noise either under a high signal-to-noise ratio (SNR; low demand) or a low SNR (high demand). In Experiment 2, speech clarity was manipulated by adding a meaningless pink noise whose energy was perfectly correlated with a sentence’s amplitude envelope to maintain constant acoustic masking throughout a sentence (Davis et al., 2011). We expect that pupil dilation will increase for acoustically and semantically challenging sentences compared with less challenging ones and that acoustic and linguistic challenges interact in their effect on pupil dilation.

## Methods and Materials

Data are publicly available at https://osf.io/9kfn4/

### Participants

Seventy-three graduate and undergraduate students from The University of Western Ontario (Canada) were recruited in two experiments (Experiment 1: *N *= 38, mean age: 20.4 years, range: 18–33 years, 26 females; Experiment 2: *N* = 35, mean age: 19 years, range: 17–21 years, 15 females). One person who participated in Experiment 1 did not provide information regarding age and sex but was recruited from the same student population. Data from one additional participant recorded for Experiment 2 were excluded due to failure in data storage. Participants self-reported having normal hearing, normal or corrected-to-normal vision, and no neurological disorders in their history. Participants gave written informed consent and received course credits or were paid $10 per hour for their participation. The experimental protocols were approved by the Research Ethics Board of the University of Western Ontario (protocol ID: HSREB 106570) and are in line with the Declaration of Helsinki.

### Auditory Stimuli and Task

We used sentence materials from previous studies, in which the effect of sentence ambiguity on behavior and on brain activity was investigated (Rodd et al., 2005, 2010a). Two conditions were used. In the high-ambiguity (HA) condition, sentences contained two or more ambiguous words (e.g., The **shell** was **fired** towards the **tank**), but the sentence meaning was not ambiguous. Sentences in the low-ambiguity (LA) condition contained no highly ambiguous words (e.g., Her secrets were written in her diary; Rodd et al., 2005). The 118 (59 HA and 59 LA) original sentences were in British English and were rerecorded by a female English speaker native to southern Ontario Canada. The duration of sentences ranged from 1.4 s to 4.8 s. The HA and LA sentences were matched on duration and psycholinguistic parameters (number of words, imageability, naturalness, and word frequency; Rodd et al., 2005).

**Figure 1.**
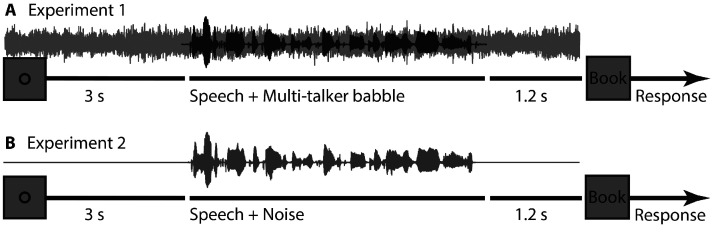
Experimental Designs for Experiments 1 and 2. Schematic timeline of a trial in Experiment 1 (A) and Experiment 2 (B). A trial started 3 s prior to sentence onset with a visual fixation ring (and in Experiment 1 with the onset of the background babble noise). A probe word was presented visually 1.2 s after sentence offset. Participants were asked to indicate whether the probe word was semantically related or unrelated to the sentence.

In Experiment 1 (Figure 1A), sentences were masked by 30-talker babble either at a low or at a high SNR. It was generated by concatenating the current set of sentences 30 times in random order and then averaging across the 30 streams (Wagner et al., 2003). Given its composition, the 30-talker babble had the same long-term frequency spectrum as the current sentence materials and a relatively flat amplitude envelope. The 30-talker babble was cut and added to target sentences such that the babble noise started 3 s before sentence onset (cf. Zekveld et al., 2019) and ended 1.2 s after sentence offset (the babble was faded in and out over 0.01 s). Starting the babble prior to sentence onset may facilitate segregation of the target from it. Because the envelope of the 30-talker babble was quite flat, whereas the amplitude envelope of speech fluctuated naturally, masking was not constant throughout a sentence but varied with the energy in the speech signal (Wagner et al., 2003; Wendt et al., 2016). The noise level was constant across HA and LA conditions, whereas the level of the sentence was adjusted to an SNR of +6 dB (high SNR) or 0 dB (low SNR). The SNRs were chosen such that comprehension would be difficult but successful (intelligibility in the range of 80%–90%).

In Experiment 2 (Figure 1B), sentences were either presented under clear conditions or with added background noise. The background noise was created uniquely for each sentence by applying the amplitude envelope of the target sentence on that trial to pink noise (1/f noise) using the Hilbert transform (30-Hz low-pass filtered; Butterworth; Davis et al., 2011). The original sentence and the sentence-specific modulated pink noise were added at an SNR of –2 dB SNR. Because the signal and masker had the same envelope, the masking level was constant over the period of the sentence. All stimuli (including clear and those with noise added) were matched in their root-mean-square intensity level.

Both experiments were 2 × 2 factorial within-subject designs (Clarity × Ambiguity [LA, HA]). For each participant, 56 LA and 56 HA sentences were randomly selected from the 59 that were available. Half of the LA (*N* = 28) and HA (*N* = 28) sentences were randomly assigned to the low SNR condition (Experiment 1: 0 dB SNR babble; Experiment 2: –2 dB SNR pink noise), whereas the other 28 LA and 28 HA sentences were assigned to the high SNR condition (Experiment 1: +6 dB SNR babble; Experiment 2: clear). Randomization was unique for each participant. In each experiment, seven sentences per condition were presented within each of four blocks (*N* = 28 trials per block) for a total of 112 (56 HA and 56 LA) sentences per person. Sentences were presented pseudorandomly such that no more than three sentences of the same ambiguity level and two sentences of the same clarity level could occur in a row. Each participant heard each sentence only once.

For each sentence, a probe word was generated that was either semantically related (50%) or unrelated (50%) to the sentence’s meaning. These probe words were used in the relatedness judgment task in which participants were required to decide whether the word was related to the meaning of the sentences (see later).

### Procedure and Data Recording

Participants were tested in a dim, quiet room. Sentences were presented over headphones (Sennheiser HD 25-SP II) using a Steinberg UR22 (Steinberg Media Technologies) external sound card. Experimental procedures were controlled using Psychtoolbox in MATLAB (v2015b, Mathworks Inc.). Prior to the main experimental procedures, the hearing threshold was determined for each participant using a method-of-limits procedure described in detail in our previous work (Herrmann & Johnsrude, 2018). This procedure entailed alternating trials of progressively increasing or decreasing 12-second long pink noise over time by 5.4 dB/s. Participants indicated when they could no longer hear the noise (progressively decreasing intensity trial) or when they started to hear the noise (progressively increasing intensity trial). Each of the progressively increasing and decreasing intensity trials were presented six times, and at the time of the button press, the corresponding sound intensity during a trial was collected. Finally, the intensities from the 12 trials were averaged to determine the individual 50% hearing threshold. In both experiments, sounds were presented at 45 dB above the individual’s threshold (sensation level).

During the experiments, participants rested their head on a chin and forehead rest (EyeLink 1000 Tower mount) facing a screen at a distance of 67 cm. Pupil area and eye movements were recorded continuously from the left eye using an integrated infrared camera (eye tracker 1000; SMI, Needham, MA) at a sampling rate of 500 Hz. Nine-point fixation was used for eye-tracker calibration (McIntire et al., 2014).

During the experiments, each trial was structured as follows. Presentation of a fixation ring (black on gray [100 100 100] RGB background) started 3 s before sentence onset, and the fixation ring remained on the screen while the sentence was presented, until 1.2 s after sentence offset. In Experiment 1, a 30-talker babble noise was presented throughout, that is, from 3 s prior to sentence onset until 1.2 s post-sentence offset (Figure 1A). In Experiment 2, no sound stimulation was administered during the 3 s prior to sentence onset and during the 1.2-s post-sentence offset period. To ensure that participants tried to comprehend each sentence, and to assess comprehension, a semantic-relatedness judgment was required after each sentence. The fixation ring on the screen was replaced by a visual probe word (e.g., ‘Book’) 1.2 s after sentence offset. Participants had to indicate with a keypress whether the probe word was semantically related or unrelated to the sentence they had heard. The word remained on screen for 3.5 s or until participants pressed the ‘related’ (left index finger) or ‘unrelated’ (right index finger) button on a keyboard, whichever came first. The screen was cleared between trials for 5 to 7 s to allow participants to rest and blink. Participants were instructed to maintain fixation and reduce blinks as long as the fixation ring was presented on the screen (including during presentation of sound materials).

Before both experiments, participants underwent a training block of eight trials (using sentences not used in the experiment) to familiarize them with the experimental procedures (including eye-tracker calibration). The experiment took approximately 1 hr to complete.

### Data Analysis

Data analysis was carried out offline using custom MATLAB scripts (v2018b), and the analyses were identical for both experiments.

#### Behavior

The semantic-relatedness responses were analyzed by calculating the proportion of correct responses, separately for each ambiguity and speech-clarity condition. A correct response entailed responding with the ‘related’ button when a word was semantically related to the preceding sentence or by pressing the ‘unrelated’ button when the word was not semantically related to the preceding sentence. Separately for each experiment, a 2 × 2 repeated-measures analysis of variance (rmANOVA) was calculated, with factors Clarity (Experiment 1: +6 dB SNR, 0 dB SNR; Experiment 2: clear, –2 dB SNR) and Ambiguity (LA, HA).

#### Pupillometry

Preprocessing of pupil area involved removing eye-blink artifacts. For each eye blink indicated by the eye tracker, all data points between 50 ms before and 200 ms after a blink were set to NaN (‘not a number’ in MATLAB). In addition, pupil area values that differed from the median pupil area by more than 3 times the median absolute deviation were classified as outliers and set to NaN (Leys et al., 2013). Missing data (coded as NaN) resulting from artifact rejections and outlier removal were linearly interpolated. Data for an entire trial were excluded from analysis if the percentage of NaN data entries made up more than 40% of the trial, ranging from 0.5 s prior to sentence onset to 1 s after sentence offset (excluded trials [mean]: Experiment 1: 1.7%, Experiment 2: 1.6%; interpolated data points in analyzed trials [mean]: Experiment 1: 1.2%, Experiment 2: 2%). Data were low-pass filtered at 10 Hz (Kaiser window, length: 201 points). Single-trial time courses were baseline-corrected by subtracting the mean pupil size from the –0.5 s to 0 s time window from the pupil size value at each time point (Mathôt et al., 2018). Single-trial time courses were averaged separately for each condition and displayed for the –0.5 s to 4 s epoch.

Three dependent measures were extracted: mean pupil dilation, peak pupil dilation, and peak pupil latency (Winn et al., 2018; Zekveld et al., 2010). To account for the different sentence durations at the analysis stage, mean pupil dilation was calculated for each trial as the average pupil area within 0.5 s post sentence onset and 1 s post sentence offset, and subsequently averaged across trials, separately for each condition and participant. Peak dilation and peak latency were extracted for each trial within 0.5 s post sentence onset and 1 s post sentence offset, and subsequently averaged across trials, separately for each condition and participant.

Separately for each experiment and each dependent measure, a 2 × 2 rmANOVA was calculated, with factors Clarity (Experiment 1: +6 dB SNR, 0 dB SNR; Experiment 2: clear, –2 dB SNR) and Ambiguity (LA, HA).

#### Microsaccades

Participants were instructed to maintain fixation and reduce blinks during a trial. Microsaccades commonly occur during prolonged fixation in auditory tasks (Widmann et al., 2014), such as was used here, and microsaccades can decrease pupil dilation (Knapen et al., 2016). We therefore tested the extent to which microsaccades show effects of speech clarity and semantic ambiguity. Microsaccades were identified using a method that computes thresholds based on velocity statistics from eye-tracker data and then identfies microsaccades as events passing that threshold (Engbert, 2006; Engbert & Kliegl, 2003). That is, the veritical and horizontal eye movement time series were transformed into velocities, and microsaccades were classified as outliers if they exceeded a relative velocity threshold of 15 times the standard deviation of the eye-movement velocity and persisted for 6 ms or longer (Engbert, 2006; Engbert & Kliegl, 2003). A time course of microsaccade rate was calculated from the individual microsaccade times (Widmann et al., 2014) by convolving each microsaccade occurrence with a Gaussian window (standard deviation of 0.02 s; zero phase lag). Mean microsaccade rate was calculated across trials as the average rate in the time window ranging from 0.5 s post sentence onset to 1 s post sentences offset, and subsequently averaged across trials (similar to the analysis of mean pupil dilation). For display purposes, time courses of mean microsaccade rate were calculated for the –0.5 to 4 s time window relative to sentence onset.

Separately for each experiment, a 2 × 2 rmANOVA was calculated for the mean microsaccade rate, with factors Clarity (Experiment 1: +6 dB SNR, 0 dB SNR; Experiment 2: clear, –2 dB SNR) and Ambiguity (LA, HA).

## Results

### Experiment 1

#### Semantic-Relatedness Task

**Figure 2.**
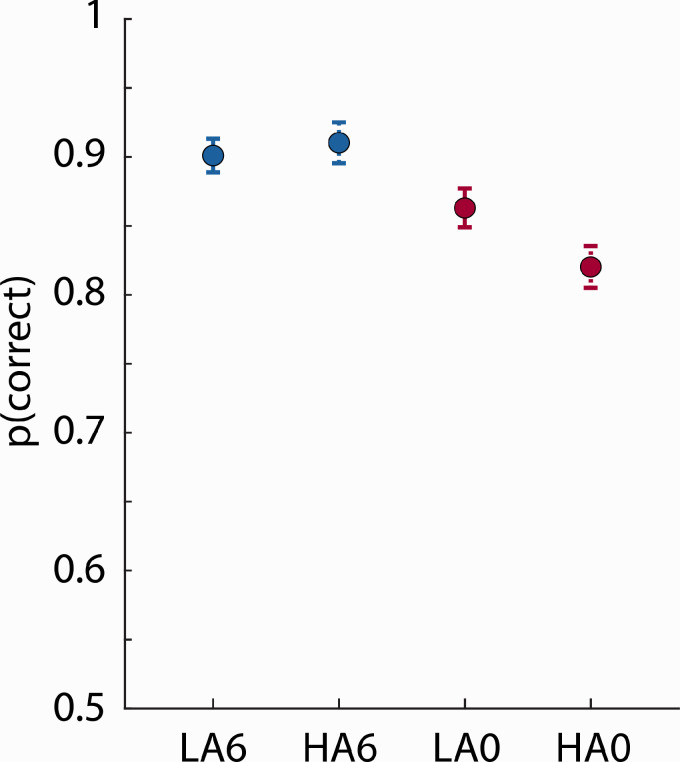
Experiment 1: Proportion Correct in Semantic-Relatedness Task. Mean proportion of correct responses for each condition. Error bars reflect the standard error of the mean. The Clarity × Ambiguity interaction was significant. LA6 = low ambiguity in +6 dB SNR babble; HA6 = high ambiguity in +6 dB SNR babble; LA0 = low ambiguity in 0 dB SNR babble; HA0 = high ambiguity in 0 dB SNR babble.

Mean proportion correct in the semantic-relatedness task was greater than 0.8 for all conditions (Figure 2). The rmANOVA on these data revealed that proportion correct was higher at +6 dB SNR than at 0 dB SNR—Clarity: *F*(1, 37) = 54.103, *p* < 1^e-8^, $\eta_{p}^{2}$ = 0.594. The main effect of Ambiguity was not significant, *F*(1, 37) = 2.698, *p* = .109, $\eta_{p}^{2}$ = 0.068, but the Clarity × Ambiguity interaction was significant, *F*(1, 37) = 8.265, *p* = .007, $\eta_{p}^{2}$ = 0.183, such that participants performed worse for HA sentences compared with LA sentences at 0 dB SNR, *F*(1, 37) = 8.355, *p* = .0066, $\eta_{p}^{2}$ = 0.184, but not at +6 dB SNR, *F*(1, 37) = 0.564, *p* = .458, $\eta_{p}^{2}$ = 0.015.

#### Pupillometry

**Figure 3.**
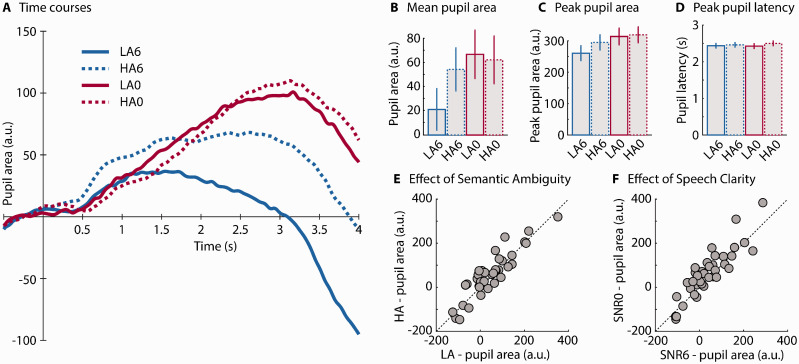
Experiment 1: Pupil Dilation Results. (A) Time course of pupil area (averaged across participants; *N* = 38). (B) Mean pupil area from 0.5 s after sentence onset to 1 s after sentence offset. (C) Peak pupil area. (D) Peak pupil latency. Error bars reflect the standard error of the mean. (E) Individual data scatterplot for Ambiguity main effect (mean pupil area). (F) Individual scatterplot for Clarity main effect (mean pupil area). LA6 = low ambiguity in +6 dB SNR babble; HA6 = high ambiguity in +6 dB SNR babble; LA0 = low ambiguity in 0 dB SNR babble; HA0 = high ambiguity in 0 dB SNR babble; SNR = signal-to-noise ratio.

Pupil area time courses are displayed in Figure 3A. The rmANOVA for the mean pupil area revealed that the pupil area was larger at 0 dB SNR than at +6 dB SNR—Clarity: *F*(1, 37) = 10.34, *p* = .003, $\eta_{p}^{2}$ = 0.218 (Figure 3B and F). In addition, pupil area tended to be larger for HA sentences compared with LA sentences—trend toward effect of Ambiguity: *F*(1, 37) = 3.73, *p* = .061, $\eta_{p}^{2}$ = 0.092 (Figure 3B and E). Individual data points are shown in Figure 3E and F; the diagonal line indicates where data would fall if there was no effect of Ambiguity (3E) or Clarity (3 F), with above the line indicating larger pupil area for HA or the lower SNR. The Clarity × Ambiguity interaction also approached significance, *F*(1, 37) = 3.91, *p* = .056, $\eta_{p}^{2}$ = 0.095. Because this interaction was hypothesized, we analyzed the simple effects and observed that pupil area was larger in HA compared with LA sentences at +6 dB SNR, *F*(1, 37) = 8.72, *p* = .005, $\eta_{p}^{2}$ = 0.191, but not at 0 dB SNR, *F*(1, 37) = 0.13, *p* = .724, $\eta_{p}^{2}$ = 0.003.

The rmANOVA for peak pupil area revealed that peak pupil dilation was larger at 0 dB SNR than at +6 dB SNR—Clarity: *F*(1, 37) = 18.11, *p* = 1.3^e-4^, $\eta_{p}^{2}$ = 0.329, and larger for HA compared with LA sentences—Ambiguity: *F*(1, 37) = 4.72, *p* = .036, $\eta_{p}^{2}$ = 0.113 (Figure 3C). The Clarity × Ambiguity interaction was not significant, *F*(1, 37) = 2.20, *p* = .147, $\eta_{p}^{2}$ = 0.056.

The rmANOVA on peak latency revealed no significant main effects—Clarity: *F*(1, 37) = 0.264, *p* = .611, $\eta_{p}^{2}$ = 0.007; Ambiguity: *F*(1, 37) = 3.486, *p* = .070, $\eta_{p}^{2}$ = 0.086—and no interaction, *F*(1, 37) = 0.537, *p* = .468, $\eta_{p}^{2}$ = 0.014 (Figure 3D).

In sum, Experiment 1 demonstrates that for masked but still highly intelligible (more than 80%) materials, pupil area is sensitive to speech clarity and semantic ambiguity, indicating that both acoustic and linguistic factors affect pupil dilation. In both conditions, a babble noise was used as the masker, which may have introduced some informational masking, likely requiring cognitive control and distracter suppression (Johnsrude & Rodd, 2016), as well as energetic masking. In Experiment 2, we used a pink noise masker with a constant SNR of –2 dB relative to the spoken sentences: This masker was used to investigate whether pupil dilation is also sensitive to linguistic factors when energetic masking is constant. This probably makes demands on working memory and requires access to semantic knowledge for effective use of context (Johnsrude & Rodd, 2016). We use clear speech as the high SNR condition to evaluate whether the effect of Ambiguity is still present on pupil responses, even when no background noise is present.

### Experiment 2

#### Semantic-Relatedness Task

**Figure 4.**
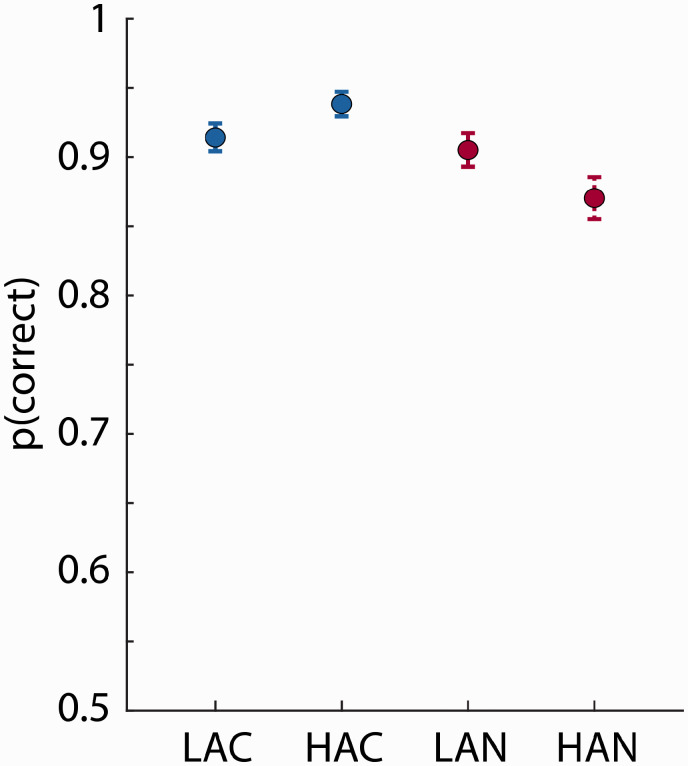
Experiment 2: Proportion Correct in Semantic-Relatedness Task. Mean proportion of correct responses for each condition. Error bars reflect the standard error of the mean. The Clarity × Ambiguity interaction was significant. LAC = low ambiguity in clear; HAC = high ambiguity in clear; LAN = low ambiguity in –2 dB SNR pink noise; HAN = high ambiguity in –2 dB SNR pink noise.

Mean proportion correct in the semantic-relatedness task exceeded 0.85 for all conditions (Figure 4). The proportion of correct responses was lower for –2 dB SNR compared with clear sentences—Clarity: *F*(1, 34) = 24.298, *p* = 2.1^e-5^, $\eta_{p}^{2}$ = 0.417. The effect of Ambiguity was not significant, *F*(1, 34) = 0.512, *p* = .479, $\eta_{p}^{2}$ = 0.015, but a significant Clarity × Ambiguity interaction, *F*(1, 34) = 6.797, *p* = .013, $\eta_{p}^{2}$= 0.167, was due to lower performance for HA compared with LA sentences at –2 dB SNR, *F*(1, 34) = 5.165, *p* = .029, $\eta_{p}^{2}$= 0.132, but higher performance for HA compared with LA for clear sentences, *F*(1, 34) = 4.427, *p* = .043, $\eta_{p}^{2}$= 0.115.

#### Pupillometry

**Figure 5.**
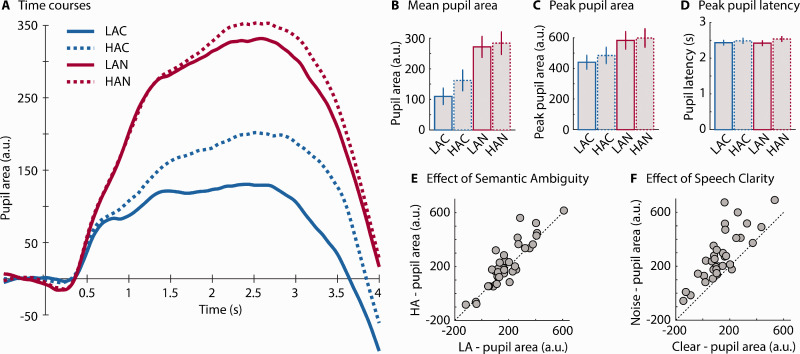
Experiment 2: Pupil Dilation Results. (A) Time course of pupil area (averaged across participants; *N* = 35). (B) Mean pupil area from 0.5 s after sentence onset to 1 s after sentence offset. (C) Peak pupil area. (D) Peak pupil latency. Error bars in (B), (C), and (D) reflect the standard error of the mean. (E) Individual data scatterplot for the Ambiguity main effect (mean pupil area). (F) Individual data scatterplot for Clarity main effect (mean pupil area). LAC = low ambiguity in clear; HAC = high ambiguity in clear; LAN = low ambiguity in –2 dB SNR pink noise; HAN = high ambiguity in –2 dB SNR pink noise.

Pupil area time courses are displayed in Figure 5A. The rmANOVA for the mean pupil area revealed that mean pupil area was larger at –2 dB SNR compared with clear sentences—Clarity: *F*(1, 34) = 55.69, *p* = 1.169^e-8^, $\eta_{p}^{2}$= 0.621 (Figure 5B and F). Mean pupil area was also larger for HA than for LA sentences—Ambiguity: *F*(1, 34) = 5.54, *p* = .025, $\eta_{p}^{2}$= 0.14 (Figure 5B and E). The Clarity × Ambiguity interaction was not significant, *F*(1, 34) = 1.80, *p* = .188, $\eta_{p}^{2}$= 0.05. Given the theoretically important question of whether pupil area differed between clear HA and clear LA sentences, we tested the simple effect of Ambiguity for Clear speech: Indeed, mean pupil area was larger for HA than LA sentences, *F*(1, 34) = 4.69, *p* = .037, $\eta_{p}^{2}$= 0.121. Individual data points are shown in Figure 5E and F; the diagonal line indicates where data would fall if there was no effect of Ambiguity (5E) or Clarity (5 F), with above the line indicating larger pupil area for HA or the –2 dB SNR.

The results for peak pupil area mirrored those for mean pupil area (Figure 5C). Peak pupil area was larger in the noise compared with the clear conditions—Clarity: *F*(1, 34) = 53.57, *p* = 1.77^e-8^, $\eta_{p}^{2}$= 0.612, and larger in the HA than in LA conditions—Ambiguity: *F*(1, 34) = 6.729, *p* = .0139, = 0.165. The Clarity × Ambiguity interaction was not significant, *F*(1, 34) = 0.2834, *p* = .283, $\eta_{p}^{2}$ = 0.034. Again, we tested whether sentence ambiguity influences pupil area when sentences were clear. Peak pupil area was larger for clear HA than clear LA sentences, *F*(1, 34) = 4.96, *p* = .033, $\eta_{p}^{2}$ = 0.127.

The rmANOVA for peak latency (Figure 5D) revealed no effect for Clarity, *F*(1, 34) = 0.222, *p* = .641, $\eta_{p}^{2}$ = 0.006, but pupil dilation peaked later for HA than for LA sentences, Ambiguity: *F*(1, 34) = 11.487, *p* = .002, $\eta_{p}^{2}$ = 0.253. There was no Clarity × Ambiguity interaction, *F*(1, 34) = 1.435, *p* = .239, $\eta_{p}^{2}$ = 0.040.

### Pooling Data From Experiments 1 and 2

To compare behavioral performance in the semantic-relatedness task across experiments, to gain more statistical power to observe any Clarity × Ambiguity interaction on pupil area, and to explore correlations between behavioral performance and pupil variables, we pooled the data from Experiments 1 and 2 (*N* = 73). We performed rmANOVAs as before, with Experiment as a between-subjects factor.

#### Semantic-Relatedness Task

Behavioral performance was higher in Experiment 2 compared with Experiment 1, *F*(1, 71) = 5.095, *p* = .0271, $\eta_{p}^{2}$ = 0.067. Behavioral performance was higher for high compared with low SNR conditions—Clarity: *F*(1, 71) = 75.909, *p* < .00001, $\eta_{p}^{2}$ = 0.517. The main effect of Ambiguity was not significant, *F*(1, 71) = 2.972, *p* = .089, $\eta_{p}^{2}$ = 0.040, but there was a Clarity × Ambiguity interaction, *F*(1, 71) = 14.905, *p* = .000247, $\eta_{p}^{2}$ = 0.174, such that performance was lower for HA compared with LA sentences at low SNRs, *F*(1, 72) = 13.561, *p* = .0004, $\eta_{p}^{2}$ = 0.158, and trended toward higher performance on HA compared with LA sentences in high-SNR conditions, *F*(1, 72) = 3.767, *p* = .0562, $\eta_{p}^{2}$ = 0.050. The effect of Clarity was larger than the effect of Ambiguity, *F*(1, 72) = 45.333, *p* < .0001, $\eta_{p}^{2}$ = 0.386. The effect of Clarity was larger in Experiment 1 compared with Experiment 2—Clarity × Experiment interaction: *F*(1, 71) = 4.701, *p* = .034, $\eta_{p}^{2}$ = 0.062, but not the effect of Ambiguity—Ambiguity × Experiment interaction: *F*(1, 71) = 0.798, *p* = .375, $\eta_{p}^{2}$ = 0.011. The three-way interaction was not significant.

#### Pupillometry

Mean pupil area was larger in Experiment 2 compared with Experiment 1, *F*(1, 71) = 26.65, *p* = 2^e-6^, $\eta_{p}^{2}$ = 0.273. Mean pupil area was larger for low SNR compared with high SNR conditions—Clarity: *F*(1, 71) = 69.774, *p* = 3.7^e-12^, $\eta_{p}^{2}$ = 0.496, and larger for HA compared with LA sentences—Ambiguity: *F*(1, 71) = 9.32, *p* = .003, $\eta_{p}^{2}$ = 0.116. The Clarity × Ambiguity interaction, *F*(1, 71) = 4.97, *p* = .029, $\eta_{p}^{2}$ = 0.066, revealed that pupil area was larger for HA compared with LA sentences at high SNRs, *F*(1, 72) = 10.73, *p* = .002, $\eta_{p}^{2}$ = 0.130, but not at low SNRs, *F*(1, 72) = 0.123, *p* = .726, $\eta_{p}^{2}$ = 0.002. The effect of Clarity was larger than the effect of Ambiguity, *F*(1, 72) = 42.901, *p* < .0001, $\eta_{p}^{2}$ = 0.373. The effect of Clarity was larger in Experiment 2 compared with Experiment 1—Clarity × Experiment interaction: *F*(1, 71) = 32.814, *p* < .00001, $\eta_{p}^{2}$ = 0.316, but not the effect of Ambiguity, Ambiguity × Experiment interaction: *F*(1, 71) = 1.395, *p* = .242, $\eta_{p}^{2}$ = 0.019. The three-way interaction was not significant.

The rmANOVA for peak pupil area mirrored the results for mean pupil area. Peak pupil area was larger in Experiment 2 compared with Experiment 1, *F*(1, 71) = 22.349, *p* = .000011, $\eta_{p}^{2}$ = 0.239. Peak pupil area was larger for low SNR compared with high SNR conditions—Clarity: *F*(1, 71) = 74.859, *p* < .00001, $\eta_{p}^{2}$ = 0.513, and larger for HA compared with LA sentences—Ambiguity: *F*(1, 71) = 11.625, *p* = .0011, $\eta_{p}^{2}$ = 0.141. The Clarity × Ambiguity interaction approached significance, *F*(1, 71) = 3.161, *p* = .080, $\eta_{p}^{2}$ = 0.043, showing that pupil area was larger for HA compared with LA sentences at high SNRs, *F*(1, 72) = 13.02, *p* = .0006, $\eta_{p}^{2}$ = 0.153, but not for low SNRs, F(1, 72) = 0.873, *p* = .353, $\eta_{p}^{2}$ = 0.012. The effect of Clarity was larger than the effect of Ambiguity, *F*(1, 72) = 53.973, *p* < .0001, $\eta_{p}^{2}$ = 0.428. The effect of Clarity was larger in Experiment 2 compared with Experiment 1—Clarity × Experiment interaction: *F*(1, 71) = 21.711, *p* = .000014, $\eta_{p}^{2}$ = 0.234, but not the effect of Ambiguity—Ambiguity × Experiment interaction: *F*(1, 71) = 0.479, *p* = .491, $\eta_{p}^{2}$= 0.007. The three-way interaction was not significant.

The rmANOVA for peak latency revealed that pupil dilation peaked later for HA than for LA sentences—Ambiguity: *F*(1, 71) = 13.519, *p* = .0005, $\eta_{p}^{2}$= 0.016. None of the other effects and interactions were significant (all *F* < 1.7, *p* > .2).

#### Correlation Between Behavioral Performance and Pupil Area

We examined whether comprehension (indexed by performance on the relatedness task) was related to pupil area by calculating correlations between behavioral performance and mean pupil area, partialing out Experiment so as to avoid biasing correlations by overall differences between experiments. No significant correlations were observed. The correlation between performance and pupil area, collapsed across clarity and ambiguity levels, was not significant (*r* = –.218, *p* = .065, *df* = 70). The correlation between the difference in HA versus LA behavioral performance and the HA versus LA difference in mean pupil area, collapsed across clarity levels, was also not significant (*r* = 0.197, *p* = .097, *df* = 70) and neither was the correlation between the difference in low SNR versus high SNR behavioral performance and low SNR versus high SNR difference in mean pupil area, collapsed across ambiguity levels (*r* = .089, *p* = .455, *df* = 70). Finally, the correlation between the HA versus LA difference in behavioral performance and the HA versus LA difference in mean pupil area was not significant in the simple effects: either at high SNRs (*r* = .117, *p* = .330, *df* = 70) nor at low SNRs (*r* = –.098, *p* = .414, *df* = 70). Thus, there appears to be no relation between mean pupil area and comprehension, at least as indexed by the semantic-relatedness task used here.

### Microsaccade Results

**Figure 6.**
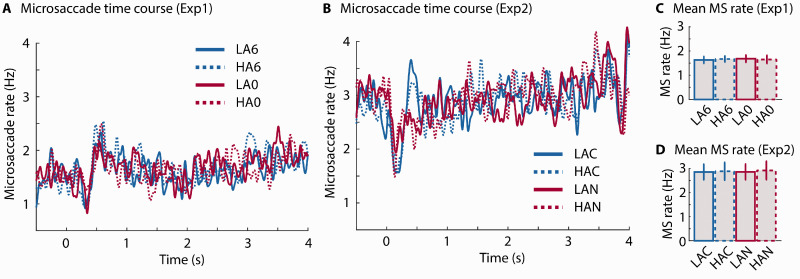
Results for Microsaccade Analysis. Time courses of microsaccade rate for Experiment 1 (A) and Experiment 2 (B). Bar graphs show the mean microsaccade rate for each condition for Experiment 1 (C) and Experiment 2 (D). Error bars reflect the standard error of the mean. MS = microsaccade; LA6 = low ambiguity in +6 dB SNR babble; HA6 = high ambiguity in +6 dB SNR babble; LA0 = low ambiguity in 0 dB SNR babble; HA0 = high ambiguity in 0 dB SNR babble; LAC = low ambiguity in clear; HAC = high ambiguity in clear; LAN = low ambiguity in –2 dB SNR pink noise; HAN = high ambiguity in –2 dB SNR pink noise.

Microsaccades were analyzed to investigate whether saccadic eye movements during fixation are also sensitive to speech clarity and semantic ambiguity. Microsaccade time courses are depicted in Figure 6. The initial decrease in microsaccade rate after sentence onset is consistent with previous work showing a transient reduction in microsaccade rate for task-relevant auditory stimuli (Widmann et al., 2014).

Microsaccade rate, averaged across the epoch spanning 0.5 s post-sentence onset to 1 s post-sentence offset, is shown in Figure 6C and D. No significant main effects or interactions were observed in the two experiments—Experiment 1: Clarity: *F*(1, 37) = 0.051, *p* = .821, $\eta_{p}^{2}$ = 0.001; Ambiguity: *F*(1, 37) = 0.003, *p* = .956, $\eta_{p}^{2}$= 8^e-5^; Clarity × Ambiguity interaction: *F*(1, 37) = 0.316, *p* = .577, $\eta_{p}^{2}$= 0.008; Experiment 2: Clarity: *F*(1, 34) = 0.039, *p* = .844, $\eta_{p}^{2}$= 0.001; Ambiguity: *F*(1, 34) = 0.122, *p* = .729, $\eta_{p}^{2}$= 0.003; Clarity × Ambiguity interaction: *F*(1, 34) = 0.017, *p* = .895, $\eta_{p}^{2}$= 5^e-4^.

An rmANOVA conducted for microsaccade data pooled across experiments was conducted, with Experiment as a between-subjects factor. The rmANOVA revealed a lower microsaccade rate in Experiment 1 compared with Experiment 2, *F*(1, 71) = 12.08, *p* = .001, $\eta_{p}^{2}$= 0.145.

## Discussion

### Speech Comprehension

In the current study, we conducted two experiments to investigate the effects of speech clarity and semantic ambiguity on sentence comprehension and pupil dilation. Speech comprehension was good throughout as indexed by a semantic-relatedness task (all scores higher than 80% correct) but was reliably lower for acoustically degraded compared with less degraded sentences in both experiments, as expected (e.g., Cherry, 1953; Johnsrude et al., 2013; Johnsrude & Rodd, 2016; Mattys et al., 2012; Miller, 1947; Ohlenforst et al., 2017).

Comprehension was also lower for sentences containing homophones than for matched sentences without, but only at the lower SNRs (0 dB but not +6 dB in Experiment 1, and with noise but not clear in Experiment 2). This is interesting given that comprehension was still high and that the two types of sentences are acoustically very similar. This effect may be due to the fact that contextual constraints are weaker in HA compared with LA sentences. Because we used meaningful sentences, their intelligibility (and thus performance on the comprehension task) is due to at least two factors. First, the acoustic quality of the signal determines intelligibility. Second, the sentence-level meaning (the context) imposes constraints that allows participants to “fill in” the words they did not hear very well, using the words that they did. In LA sentences, each of the content words has one meaning, and these meanings can constrain interpretation. Listeners can use the words they perceive from acoustically degraded LA sentences to generate a relatively small set of hypotheses regarding the identity of segments that they hear less well and then “choose to hear” words that fit with the overall meaning of the sentence. This process is less constrained for HA sentences, in which homophones are semantically consistent with a wider set of hypotheses regarding the identity of less-well-heard sentence segments. Our observation of reduced comprehension by the presence of homophones is consistent with prior work indicating that homophones in naturalistic sentences introduce increased cognitive load (compared with matched sentences without homophones) as indexed by (a) longer reaction times on a concurrent case-judgment task (Rodd et al., 2010a); (b) greater activity in functional magnetic resonance imaging experiments (Rodd et al., 2005, 2010b, 2012, 2015); and (c) poorer recognition memory (Koeritzer et al., 2018). This is the first demonstration that even when intelligibility is generally high (as evidenced by >80% accuracy on the semantic-relatedness task used here), everyday, naturalistic sentences containing ambiguous words are less well comprehended when presented with background noise, compared with sentences without such words.

### Pupillometric Measures

Pupil dilation, measured both as average area and peak area during sentence listening, was enhanced for acoustically degraded compared with less degraded sentences. This finding is in line with several previous observations demonstrating an enhanced pupil size when individuals listen under acoustic challenges (Koelewijn et al., 2014; Miles et al., 2017; Wendt et al., 2016; Winn et al., 2015; Zekveld & Kramer, 2014; Zekveld et al., 2010). Acoustic degradation due to auditory peripheral damage is associated with similar effects on pupil dilation during speech comprehension: It is larger for older compared with younger adults (Ayasse & Wingfield, 2018), for older adults with hearing loss compared with those without (Ayasse & Wingfield, 2018; but see Koelewijn et al., 2017; Wang et al., 2018), and for people with cochlear implants compared with people without (Winn, 2016).

Previous work and our findings suggest that different types of acoustic challenges all lead to enhanced pupil size. Degradation of the speech signal using noise vocoding (Winn, 2016), stationary noise (Ohlenforst et al., 2018; Zekveld et al., 2010), fluctuating noise (Koelewijn et al., 2014; Wendt et al., 2018), a single talker (Koelewijn et al., 2014; Wendt et al., 2018), multitalker babble (Ohlenforst et al., 2018; Wendt et al., 2016, 2018; current Figure 3), or noise correlated with a sentence’s amplitude envelope (current Figure 5), all increase pupil dilation relative to less-demanding control stimuli. However, just because the pupillary manifestation is similar across challenges does not mean that the cognitive resources being recruited are the same. As reviewed in the Introduction section, different demands probably recruit different processes (Johnsrude & Rodd, 2016).

The pupil was larger and peaked later when participants listened to everyday, naturalistic, sentences containing homophones compared with matched sentences without homophones. This is in line with the observation that pupil dilation increases for isolated words that are presented in the context of lexical competitors (Kuchinsky et al., 2013) or are otherwise semantically difficult to process (based on word frequency, familiarity, naming latency, and age of acquisition; Chapman & Hallowell, 2015; Kuchinke et al., 2007) compared with control words. Moreover, sentences in which semantic context does not predict the sentence’s final word lead to larger pupil dilation compared with sentences with a final word more predicable from context (Winn, 2016). Other work has demonstrated that pupil dilation increases when individuals listen to syntactically complex sentences compared with less complex ones (Ayasse & Wingfield, 2018; Wendt et al., 2016; but see Müller et al., 2019). Consistent with Kahneman’s early assertion (Kahneman, 1973; Kahneman & Beatty, 1966) that anything involving mental effort increases pupil dilation, these previous observations and our data show that not just the quality of the speech signal, but the cognitive/linguistic demands of the speech signal increase pupil dilation. This is the case even when behavioral performance is unaffected (recall that comprehension performance did not differ between HA and LA sentences when these were presented clearly [Experiment 2] or at a higher SNR [Experiment 1]).

In addition to consistent main effects of clarity and ambiguity on pupil dilation, the Clarity × Ambiguity interaction was significant for mean pupil dilation and trended toward significance for peak pupil dilation when data from both experiments were combined (but not for Experiments 1 and 2 separately). The difference in pupil response for HA compared with LA sentences was larger when signal quality was better, compared with when it was poorer (Figures 3 and 5). That the combined acoustic and linguistic challenges do not increase pupil dilation much beyond the acoustic challenge alone is consistent with the suggestion that pupil dilation approaches an asymptote for degraded, but still-intelligible speech (Ohlenforst et al., 2017, 2018; Zekveld et al., 2014, 2019). The pupil area in the current study may have approached a physiological asymptote such that, in fact, the different cognitive processes recruited to compensate for degraded speech, and to cope with the presence of homophones, may affect the pupil concurrently. Consistent with this, the pupil area was significantly larger in Experiment 2 when HA sentences were presented clearly compared with when LA sentences were presented clearly. This indicates that Ambiguity does indeed affect the pupil, even in the absence of background noise. Furthermore, others have demonstrated that pupil sizes were larger when acoustic and linguistic challenges were present concurrently than when either acoustic or linguistic challenges were presented alone (Kuchinsky et al., 2013; late time window in Wendt et al., 2016).

### Relation Between Behavioral Performance and Pupil Dilation

Comprehension behavior and pupil dilation appear to provide different windows on speech processing. At higher levels of clarity (+6 dB SNR in Experiment 1; clear presentation in Experiment 2), behavioral performance did not differ between HA and LA sentences (or performance was even somewhat higher for HA sentences; Experiment 2), whereas pupil area was larger for HA compared with LA sentences even when these were presented clearly. In contrast, at lower levels of clarity (0 dB SNR babble in Experiment 1, –2 dB SNR pink noise in Experiment 2), comprehension was reduced for HA compared with LA sentences, but the additive effect of Ambiguity on pupil area was not significant. Moreover, comprehension was generally lower in Experiment 1 compared with Experiment 2, but the absolute magnitude of the pupil area (relative to pre-sentence baseline), indexing challenges/effort, was also smaller in Experiment 1 than in Experiment 2. Furthermore, the effect of clarity level on comprehension was larger in Experiment 1 (+6 dB vs. 0 dB SNR in babble) than in Experiment 2 (clear vs. –2 dB SNR pink noise), but the effect of clarity level on pupil dilation was smaller in Experiment 1 than in Experiment 2. Hence, behavioral (comprehension) and pupil area effects of cognitive demand seem to be at least partially independent.

Although pupillometry recordings are increasingly used as a measure of listening effort (Winn et al., 2018; Zekveld et al., 2018), our data complement other results indicating that pupillometric measures do not always correlate with task performance measures or other measures of listening effort, such as subjective ratings or oscillatory neural activity (Alhanbali et al., 2019; Hicks & Tharpe, 2002; Koelewijn et al., 2012; Mackersie & Cones, 2011; Miles et al., 2017; Strand et al., 2018; Winn et al., 2015; Zekveld et al., 2010, 2019). Part of the inconsistency may be due to the fact that the term *listening effort* is ambiguous (Herrmann & Johnsrude, 2020) because it may refer to a mental act—associated with the recruitment of resources (Peelle, 2018; Pichora-Fuller et al., 2016)—or to a subjective experience (Herrmann & Johnsrude, 2020; Johnsrude & Rodd, 2016; Lemke & Besser, 2016). Different measures most certainly differ in the extent to which they tap into resource recruitment and/or experience, making the absence of correlations between behavioral performance measures and physiological measures, as well as the absence of correlations among physiological measures, less surprising.

### Microsaccades Are Not Influenced by Semantic Ambiguity and Speech Clarity

In the current experiments, participants were instructed to maintain fixation and reduce blinks during a trial. Microsaccades commonly occur during fixation (Engbert, 2006; Martinez-Conde et al., 2013; Widmann et al., 2014) and can influence pupil dilation (Knapen et al., 2016). Hence, microsaccades could in principle be entangled with changes in pupil size.

Here, we observed a transient inhibition in microsaccade rate following sentence onset (Figure 6). This is in line with previous observations that the probability of microsaccades is reduced following the onset of task-relevant auditory and visual stimuli (Rolfs et al., 2005, 2008; Widmann et al., 2014). Microsaccade inhibition is typically followed by an overshoot and a return to baseline (Rolfs et al., 2008; see also Figure 6). Critically, neither signal quality (clarity factor) nor the presence of homophones (ambiguity factor) affected microsaccade rate. The changes in pupil dilation induced by speech clarity and semantic ambiguity are therefore probably not related to microsaccades.

Analysis of microsaccade differences between experiments shows that the microsaccade rate was overall lower in Experiment 1 compared with Experiment 2 (Figure 6). Microsaccade rate has been shown to decrease with high cognitive load (Dalmaso et al., 2017; Xue et al., 2017) and task difficulty (Siegenthaler et al., 2014). This is in line with the overall lower performance in Experiment 1 compared with Experiment 2 but is in contrast to the overall larger pupil size (relative to baseline) and larger effect of speech clarity in Experiment 2 compared with Experiment 1. These results are consistent with the observation that different measures of listening effort and cognitive load are not (or only minimally) correlated (Alhanbali et al., 2019; Miles et al., 2017).

## Conclusions

The current study investigated the effects of acoustic degradation and semantic ambiguity on sentence comprehension and pupil dilation. Sentence comprehension, as indexed by performance on a semantic-relatedness task, was generally high but was reduced by masking and by semantic ambiguity. Pupil dilation increased when SNR was relatively low, and when homophones were present in everyday, naturalistic sentences, even when these were presented clearly. The current results reinforce the idea that many different challenges to speech comprehension, that afford different cognitive processes and are met by the brain in different ways, manifest as an increase in pupil dilation. When using pupillometry to measure listening effort specifically, other forms of *mental effort*, such as linguistic and domain-general abilities required to comprehend speech, and recruited only insofar as the speech signal requires them, must be controlled.
